# The Plant Health Monitoring System of the EDEN ISS Space Greenhouse in Antarctica During the 2018 Experiment Phase

**DOI:** 10.3389/fpls.2019.01457

**Published:** 2019-11-22

**Authors:** Conrad Zeidler, Paul Zabel, Vincent Vrakking, Markus Dorn, Matthew Bamsey, Daniel Schubert, Antonio Ceriello, Raimondo Fortezza, Domenico De Simone, Cecilia Stanghellini, Frank Kempkes, Esther Meinen, Angelo Mencarelli, Gert-Jan Swinkels, Anna-Lisa Paul, Robert J. Ferl

**Affiliations:** ^1^EDEN Research Group, Institute of Space Systems, Department of System Analysis Space Segment, German Aerospace Center (DLR), Bremen, Germany; ^2^Navigation and Science Organisation Unit, Telespazio S.p.A, Naples, Italy; ^3^Greenhouse Horticulture Unit, Wageningen University & Research, Wageningen, Netherlands; ^4^UFSpaceplants Lab, Horticultural Sciences Department, University of Florida, Gainesville, FL, United States

**Keywords:** EDEN ISS, plant health monitoring, greenhouse, space analogue, Antarctica, life support system, remote support, Neumayer Station III

## Abstract

The EDEN ISS project has the objective to test key technologies and processes for higher plant cultivation with a focus on their application to long duration spaceflight. A mobile plant production facility was designed and constructed by an international consortium and deployed to the German Antarctic Neumayer Station III. Future astronaut crews, even if well-trained and provided with detailed procedures, cannot be expected to have the competencies needed to deal with all situations that will arise during a mission. Future space crews, as they are today, will be supported by expert backrooms on the ground. For future space-based greenhouses, monitoring the crops and the plant growth system increases system reliability and decreases the crew time required to maintain them. The EDEN ISS greenhouse incorporates a Plant Health Monitoring System to provide remote support for plant status assessment and early detection of plant stress or disease. The EDEN ISS greenhouse has the capability to automatically capture and distribute images from its suite of 32 high-definition color cameras. Collected images are transferred over a satellite link to the EDEN ISS Mission Control Center in Bremen and to project participants worldwide. Upon reception, automatic processing software analyzes the images for anomalies, evaluates crop performance, and predicts the days remaining until harvest of each crop tray. If anomalies or sub-optimal performance is detected, the image analysis system generates automatic warnings to the agronomist team who then discuss, communicate, or implement countermeasure options. A select number of Dual Wavelength Spectral Imagers have also been integrated into the facility for plant health monitoring to detect potential plant stress before it can be seen on the images taken by the high-definition color cameras. These imagers and processing approaches are derived from traditional space-based imaging techniques but permit new discoveries to be made in a facility like the EDEN ISS greenhouse in which, essentially, every photon of input and output can be controlled and studied. This paper presents a description of the EDEN ISS Plant Health Monitoring System, basic image analyses, and a summary of the results from the initial year of Antarctic operations.

## Introduction

Bioregenerative life support systems have the potential to close the air, food, and water loops as well as provide psychological benefit to long duration space mission crews (Haeuplik-Meusburger et al., 2014; Anderson et al., 2018). Present on-orbit plant growth systems are of relatively small-scale (Massa et al., 2016). As crews venture further from Earth and for longer duration missions, larger-scale bioregenerative systems will become more important. Earth-based bioregenerative life support system test-beds serve as means to trial larger-scale and fully integrated systems, in particular with a focus on increasing the reliability of such systems. These ground-based tests also provide a means to evaluate the crew time necessary to operate these facilities while at the same time exploring possible avenues to reduce overall crew time requirements.

Both telemetry (environmental and system) and imagery data will be important in the automatic and expert backroom monitoring of future space-based plant production systems (Knott and Sager, 1991). Crop health imaging systems will play an important role in the optimization of the plant growth environment *via* the collection of biologically relevant feedback data. Essentially, the plants themselves will be queried to assess their health, and the environment adjusted accordingly or the on-site operator requested to implement other corrective actions. Various imaging techniques exist for such plant health monitoring systems (Chaerle and Van Der Straeten, 2001; Li et al., 2014; Saglam et al., 2019) and considering that controlled environment agriculture (CEA) systems can largely regulate each photon of input light, there are numerous new techniques in the exploratory phase (Ferl et al., 2017; Beisel et al., 2018).

The EDEN ISS project involved the design, development, and subsequent deployment of a containerized plant production system to the German Antarctic research station, Neumayer Station III (NM-III) (Zabel et al., 2016; Vrakking et al., 2017; Zabel et al., 2017; Schubert et al., 2018). The EDEN ISS greenhouse includes a wide array of sensors and cameras to collect data, which is subsequently made available over a satellite link to a network of backroom experts monitoring the greenhouse systems and plants. Although the facility includes an on-site operator who visits the facility for system maintenance and for horticultural management tasks, the collected data allows for a wide amount of remote operations to be conducted in an analogous means to how future space-based plant production systems will be operated.

## Eden ISS Mobile Test Facility Infrastructure

The EDEN ISS Mobile Test Facility (MTF) consists of two custom 20-ft. high-cube shipping containers. The two container approach was necessary, because the logistics chain from Germany to the Neumayer Station III in Antarctica is based on that format. The need for two containers lent itself to a natural division of the facility into a plant cultivation container, the Future Exploration Greenhouse (FEG), and a container for the supporting technical systems, the Service Section (SES), each with a distinct climate. In that way an optimal environment for plant growth can be provided in the FEG, while a decent working environment for the on-site operator can be obtained in the SES (lower CO_2_ and relative humidity levels) (Vrakking et al., 2017; Zabel et al., 2017).

The SES container is the main access point for crew entering the facility and provides space to change from winter-gear to work clothes suitable for use in the greenhouse. Additionally, most of the subsystems needed for successful plant cultivation are integrated into this container, such as the Nutrient Delivery System and the Command and Data Handling System. The FEG provides the space for actual plant cultivation and houses those components that need to be in close proximity to the plants, such as the water-cooled LED panels of the Illumination System, fans, and various sensors. The four channels (blue, red, white, and far red) of the LED panels are individually controllable and set to the specific needs of the plants. Detailed descriptions of the designs of both the SES and the FEG container have been published previously (Vrakking et al., 2017; Zabel et al., 2017).

The two containers were delivered to Antarctica in January 2018 and were shortly thereafter installed on an elevated platform situated 400 m from the German Neumayer Station III. As part of the project, one project member joined the overwintering crew at the Neumayer Station III in 2018 and acted as the on-site operator of the facility during the initial 1-year space analogue mission, carrying out the necessary nominal operational tasks, e.g., sowing/harvesting, plant growth monitoring or subsystem management, and off-nominal maintenance tasks, as well as performing various scientific tasks, e.g., sample preparation for off-line analysis.

This project member had expert knowledge of the technical aspects of the system and had received training on the plant handling and science aspects. Nevertheless, the operator in the Antarctic could not necessarily have the required knowledge and expertise to handle all possible operational situations. In particular, expertise concerning plant cultivation and the detection of disease or abnormal development in plants was identified as a critical risk to the successful operation of the greenhouse. This is similar to the situation of astronauts in space missions. The astronauts, even if well trained, cannot be experts of all domains and usually do their job according to well-defined procedures and plans with the support of ground teams of experts. The provision of the ability of these teams to monitor the status of the systems, *via* telemetry or *via* other means such as imagery, represents a way to reduce the needed crew time and responsibility of the astronauts. For this reason, additional hardware was installed in the FEG to monitor the development of the plants over time as part of the Plant Health Monitoring System, and support infrastructure was built up to enable data exchange and communication with remote experts at the various consortium partner sites.

**Figure 1:**
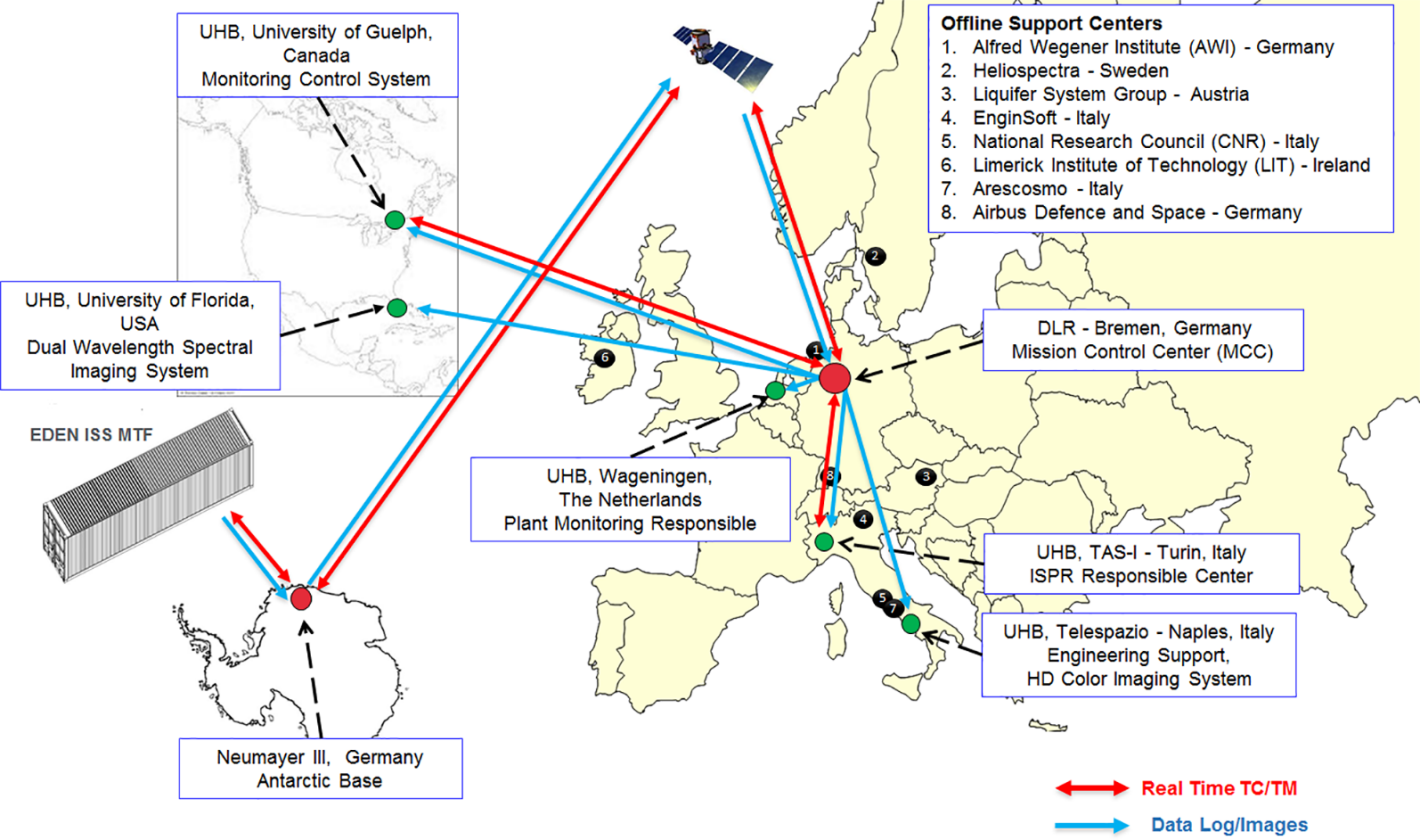
Top-level data streams in the EDEN ISS remote operations network including the MCC, the UHBs, and the offline support centers.

The remote sites were divided into six science support backrooms called User Home Bases (UHB) and the Mission Control Center (MCC) at the German Aerospace Center (DLR) in Bremen, and each site was provided with systems and tools to receive EDEN ISS data and images for real-time support and for remote commanding. The remote operations network (see **Figure 1**) was composed of:

*MCC—German Aerospace Center* (DLR—Bremen, Germany) was responsible for the overall management of EDEN ISS operations and accommodates the MCC, i.e., a control room equipped with all the tools for the remote monitoring and control of the greenhouse (including the Plant Health Monitoring System) (Schubert et al., 2018). DLR received, stored, and distributed all the data and images coming from the EDEN ISS MTF and continues to do so for current and future operation phases.

*UHB—University of Wageningen* (WUR—Wageningen, The Netherlands) was responsible for the definition of the cultivation plan (Dueck et al., 2016; Meinen et al., 2018). The WUR UHB processed and analyzed the images of the High-Definition (HD) Color Imaging System to detect anomalies and evaluate crop performance. In addition, it supported all crop cultivation-related issues.

*UHB—University of Florida* (UF—Gainesville, USA) was responsible for the Dual Wavelength Spectral Imaging System as part of the Plant Monitoring System. In this role, it analyzed the spectral images and interpreted the results for plant health and status assessment. UF was also responsible for the deposition of dual wavelength spectral images at the NASA Life Sciences Data Archive (LSDA). This was configured as a separate data transfer from UF to Kennedy Space Center (i.e., the LSDA). UF continues to fulfill these tasks for the 2019 operations phase and possibly for future phases as well.

*UHB—Telespazio* (TPZ—Naples, Italy) was responsible for the HD Color Imaging System, including development of a custom data distribution software application. As part of this responsibility, it hosted the camera test platform to provide the needed support for system configuration and troubleshooting. It was configured as a UHB for image reception and image quality assessment.

*UHB—Thales Alenia Space Italia S.p.A.* (TAS-I—Turin, Italy) was responsible for the International Standard Payload Rack (ISPR) operations. It was configured as a UHB not only to receive telemetry data and images, but was also provided with commanding capabilities to directly interact, upon MCC authorization, with the ISPR rack.

*UHB—University of Guelph* (UoG—Guelph, Canada) was responsible for the Nutrient Delivery System and support for the EDEN ISS control system. UoG was configured as a UHB with the monitoring and commanding capabilities to interact with such systems as necessary.

The MCC and UHBs provided both regularly scheduled and as-needed support to the on-site operator, resulting in successful plant cultivation and harvest of roughly 270 kg of fresh edible biomass in the first year of EDEN ISS operations (Zabel et al., 2019).

## Camera System Inside the Future Exploration Greenhouse

Imaging within the FEG was considered to be a high priority element of the monitoring of plant growth and health within the EDEN ISS project. The images provided a wide variety of flexible datasets, which could be parsed and analyzed through the system of science support backrooms to provide metrics such as leaf size and color, plant morphology and development, as well as leaf reflectance values to augment assessments of plant health. This extensive imaging collection schema is composed of two camera systems, coordinated through a central camera network imaging server. The two systems are the HD Color Imaging System, which is a collection of remote controllable, 4 MP, Red-Green-Blue (RGB) web cameras tasked with monitoring the entire FEG, and the Dual Wavelength Spectral Imagers, which are modified GoPro cameras taking images at 12 MP tasked to examine the applicability of differential wavelength imaging to plant health monitoring.

**Figure 2:**
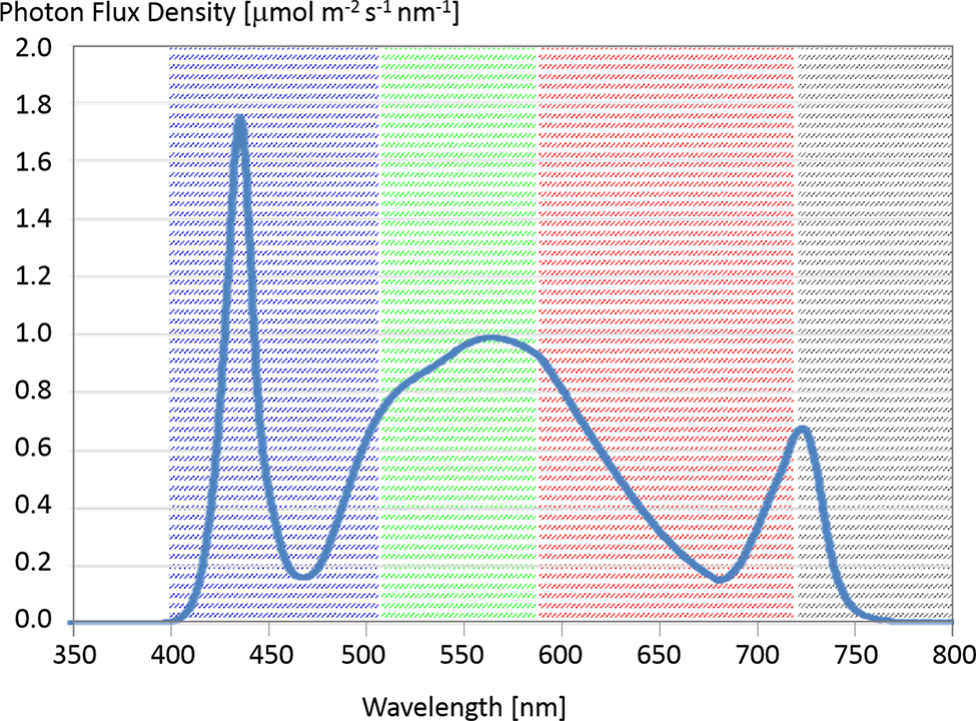
Spectral flux density (at 15-cm distance) of a LED panel set to 0% blue, 0% red, 100% white, 100% far red. There can be variation in spectral composition ±5% between individual LED panels (Source Heliospectra). The highlighted wavebands give roughly the extent of the blue, green, and red channels in the camera. The gray area shows the cut-off of the internal Near IR (NIR) filter, which was removed in the GoPro, to make it sensitive to that part of the spectrum.

The FEG offers fine control of the wavelengths of light that illuminate the plant canopies and the ability to coordinate imaging under various lighting conditions. Therefore, it is possible for both camera systems to take daily images under optimal lighting conditions for analyzing the plant status. Shortly before night mode of the greenhouse, the nominal lighting mode of the Illumination System (Zabel et al., 2019) is paused for a few minutes and switched to a special image taking mode (0% blue; 0% red; 100% white and 100% far red) to have always the same lighting conditions while taking images. The corresponding spectrum is shown in **Figure 2**.

**Figure 3:**
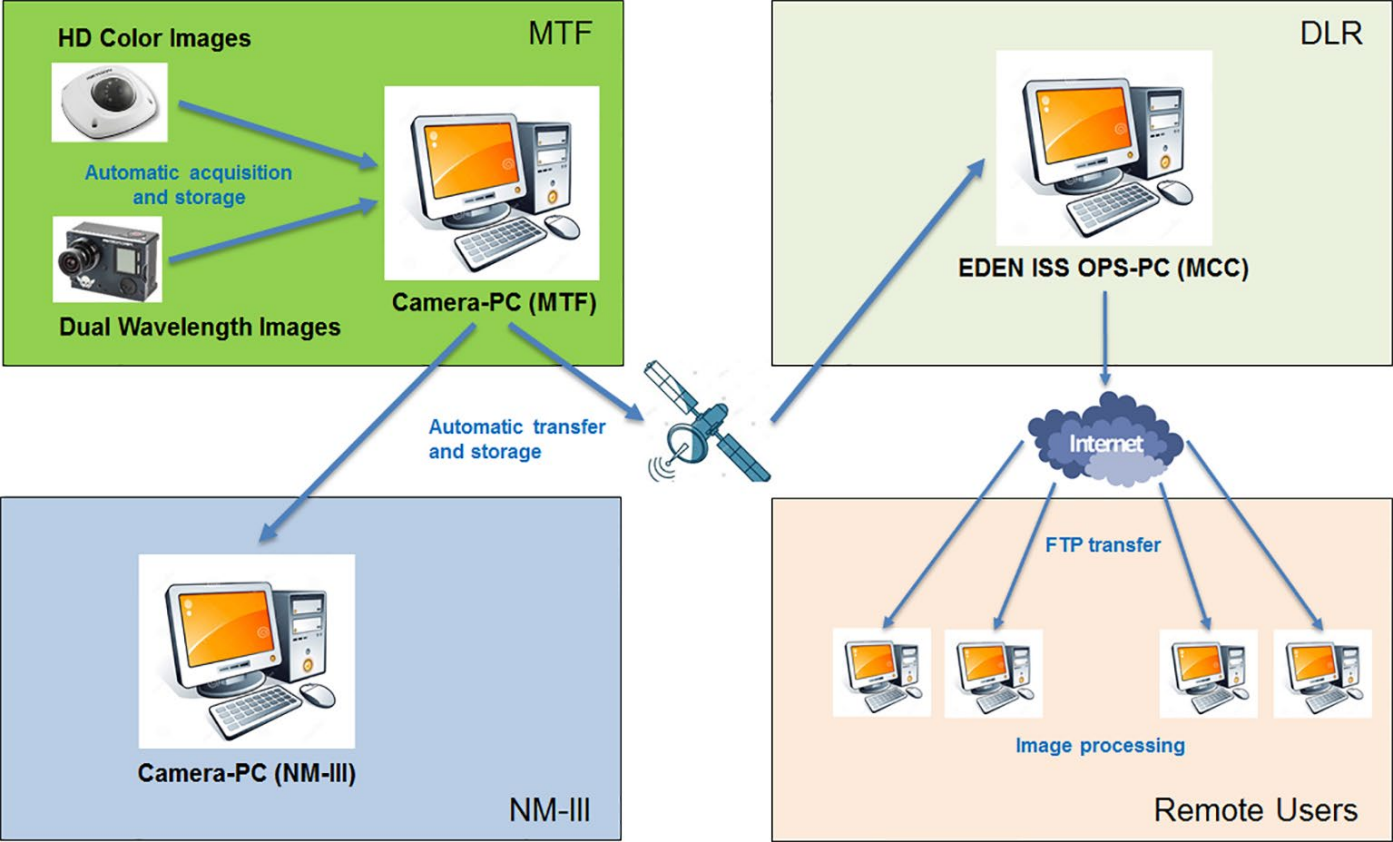
Pictorial schematic of the image acquisition and distribution process.

The images taken during image taking mode are then saved on the camera control PC in the SES and transferred during night to an FTP server in the MCC at the DLR site in Bremen and to another FTP server on a computer in the Neumayer Station III where a backup image repository is maintained. This is done by a custom developed software application based on the programming language Python. **Figure 3** provides a pictorial view of the image acquisition and distribution flow.

The primary customers of the images for initial analysis include the University of Wageningen and the University of Florida, and a large subset of the images is made available to the public for outreach purposes *via* a specially designed website. The University of Wageningen processed the images using dedicated software to assess the status of the plants. The University of Florida processed various differential images from the Dual Wavelength Spectral Imagers and forwards reports to DLR and to the LSDA.

Other project participants also had access privileges to download the images for other purposes, for example, Telespazio did so in its role of being responsible of the HD Color Imaging System.

### Description of the HD Color Imaging System

**Figure 4:**
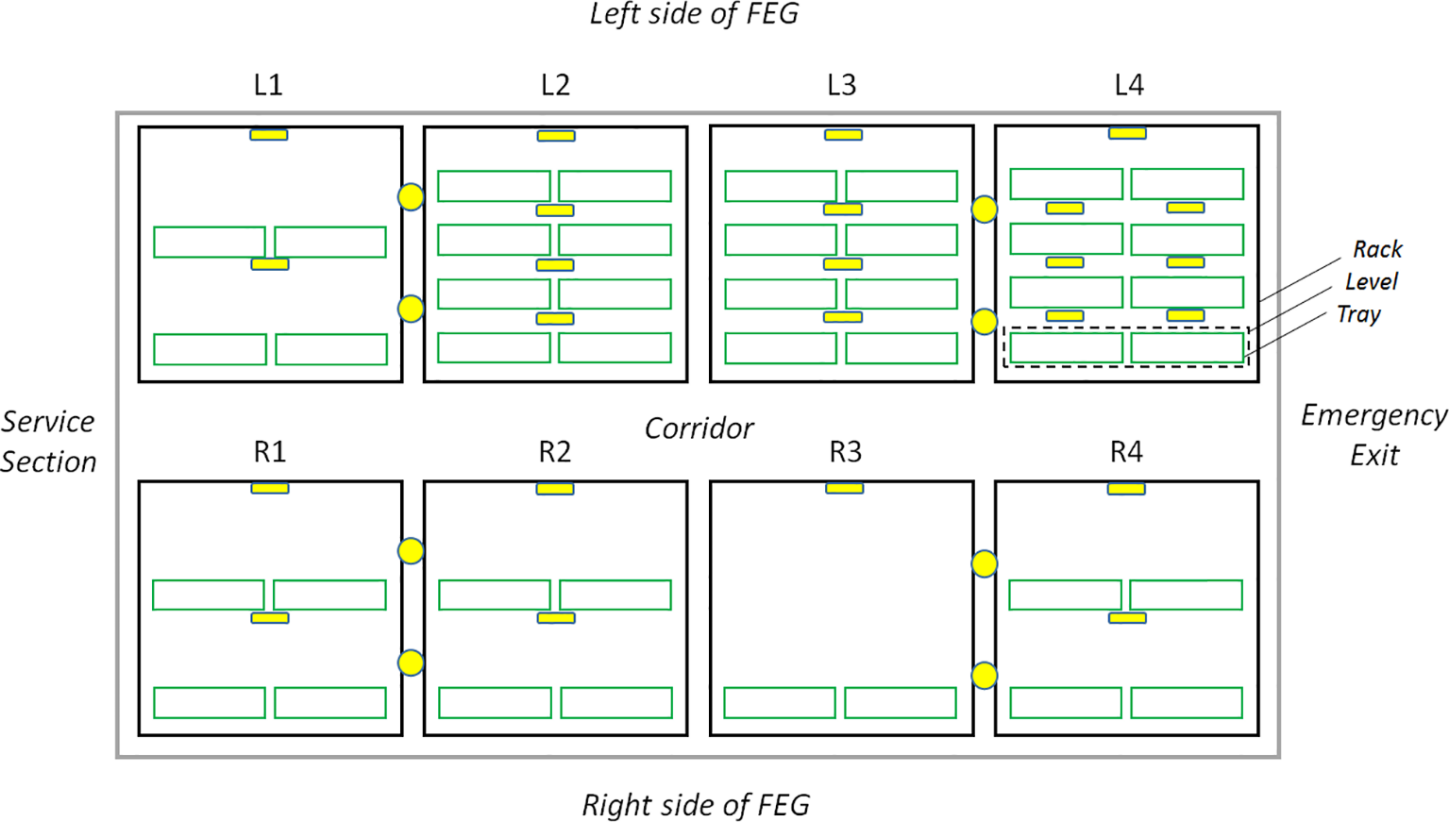
Layout of the HD Color Imaging System inside the FEG. The yellow circles represent the lateral view cameras and the yellow squares the top view cameras. Four plant growth racks (black outline) are present on each side of the container, called L1 to L4 for the left side and R1 to R4 on the right side. Each rack is subdivided into up to four growth levels (green outline) consisting of two trays for plant cultivation.

**Figure 5:**
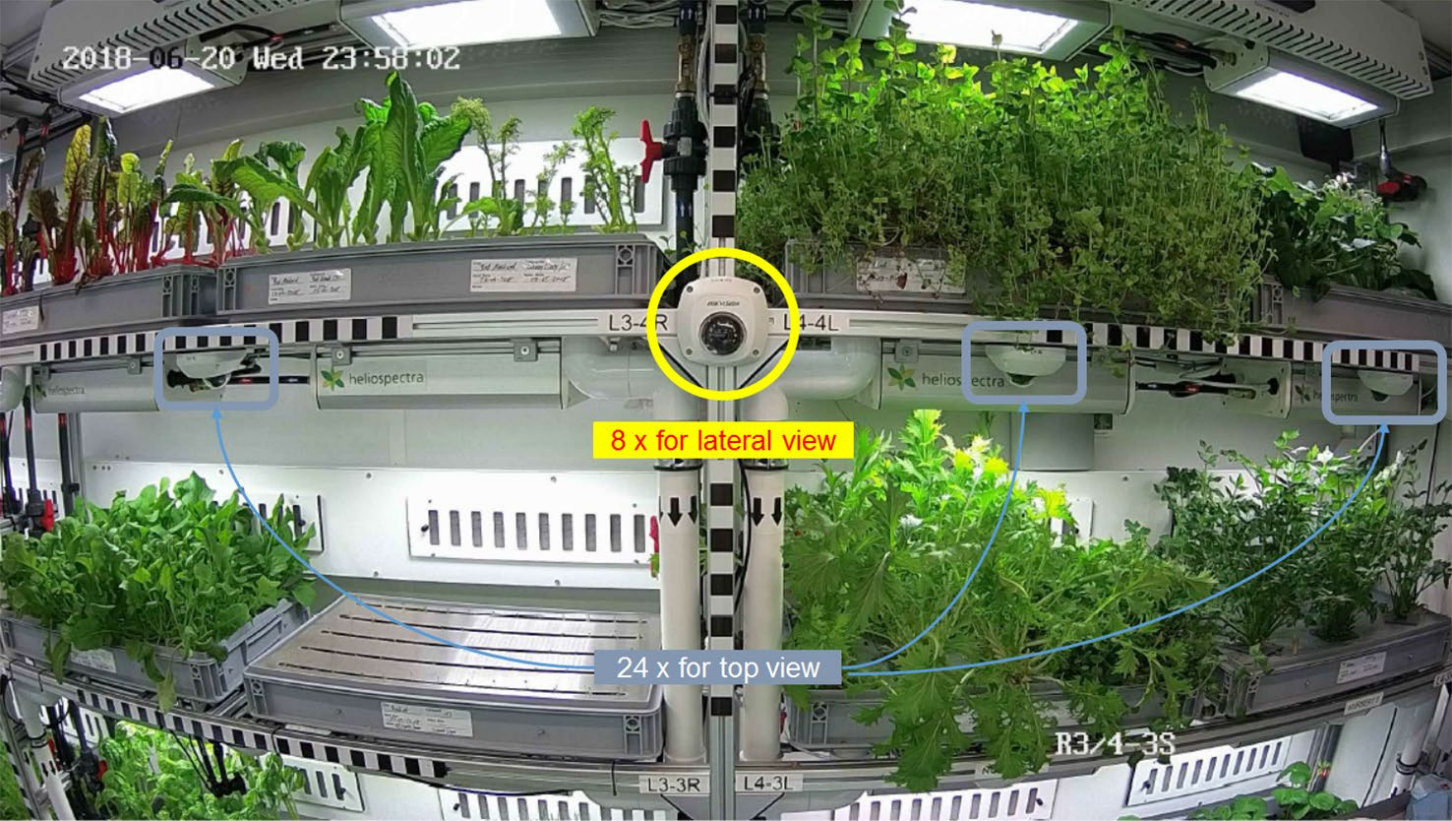
HD Color Imaging System configuration shown in a lateral view image.

**Figure 6:**
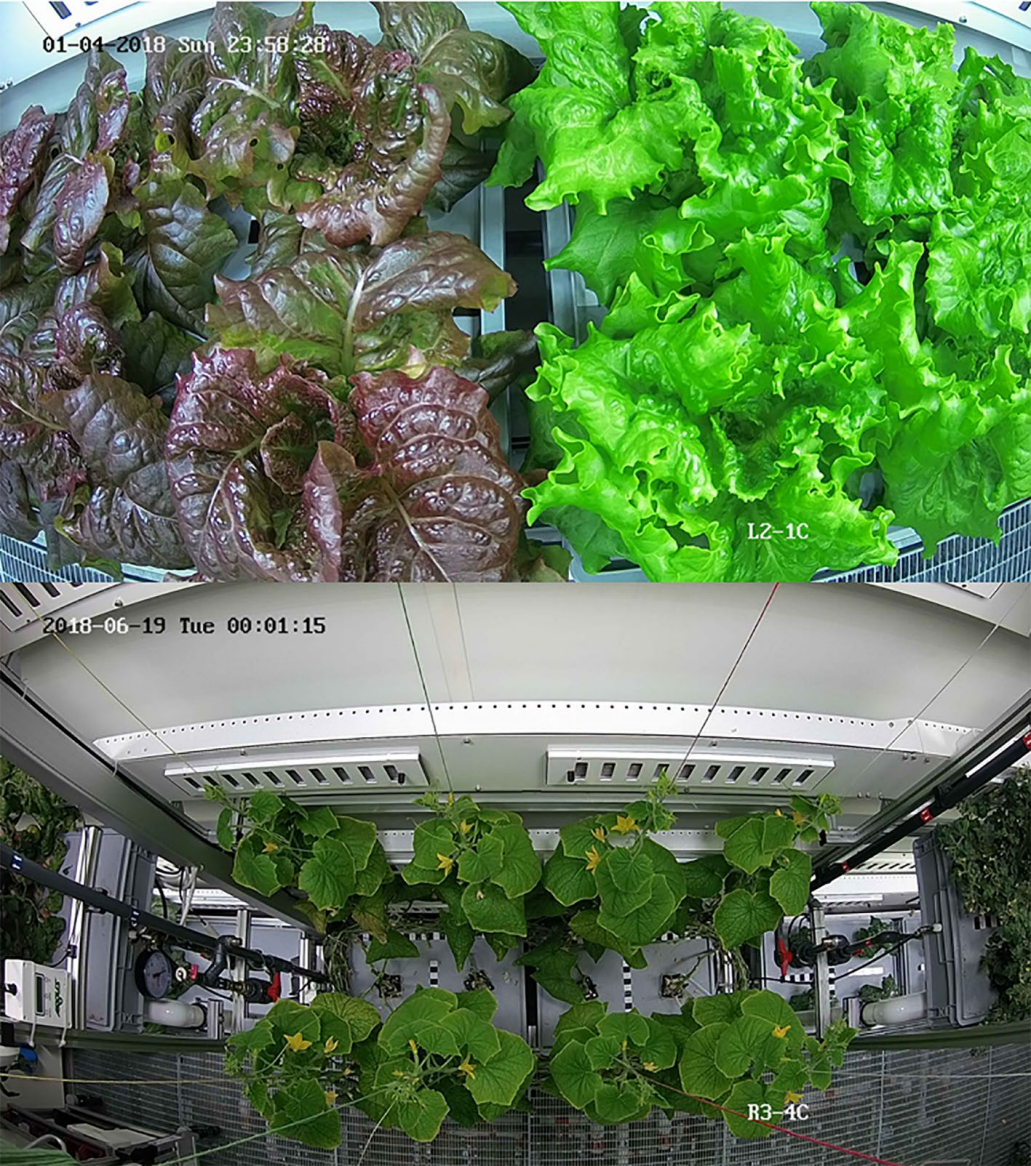
Top view images of lettuce plants (top) and cucumber plants (bottom).

The above-mentioned software of the University of Wageningen employed the images taken by the HD Color Imaging System to provide a forecast of the harvest date, for the early detection of possible problems, and to define corrective actions in the early stages of the plant health issues. For that reason, the HD Color Imaging System of the FEG is composed of several elements (see **Figures 4** and **5**):

1. Fixed system of 17 visual HD cameras, mounted on the ceiling of each rack level (with the exclusion of the nursery), pointing downward towards two trays for top view imaging (Zabel et al., 2017) (see **Figure 6**).
2. Fixed system of seven visual HD cameras mounted on the ceiling of the first to the third levels of the nursery rack, two per level/one for each tray (for the upper level, one camera is used for the whole level), pointing downward towards the trays, for top view imaging.
3. Fixed system of eight visual HD cameras, two for each rack, mounted along the corridor, on the rack structure pointing at the opposite rack for lateral view imaging (see **Figure 5**).
4. System to connect the cameras to the EDEN ISS local area network.
5. Software application for daily image acquisition, local storage, and forwarding to the European sites.

The installed cameras were the following model: HIKVISION DS-2CD2542FWD-I 4MP. The image resolution was 2,688 × 1,520 pixels and the focal length of the lens was 2.8 mm with a 106° angle of view.

The HD cameras are connected to the MTF network *via* Ethernet switches located in the FEG and the SES. In addition, they are configured by means of a computer located in the SES itself, using a dedicated camera management software program provided by HIKVISION. The camera configuration can also be done by remote sites, using the same software program installed on the NM-III and the EDEN ISS MCC camera control computers. This system provides several capabilities to the EDEN ISS operator on site and to the remote users. For example, it provides the possibility to have continuous streaming of images and to record videos and take snapshots whenever required.

**Figure 7:**
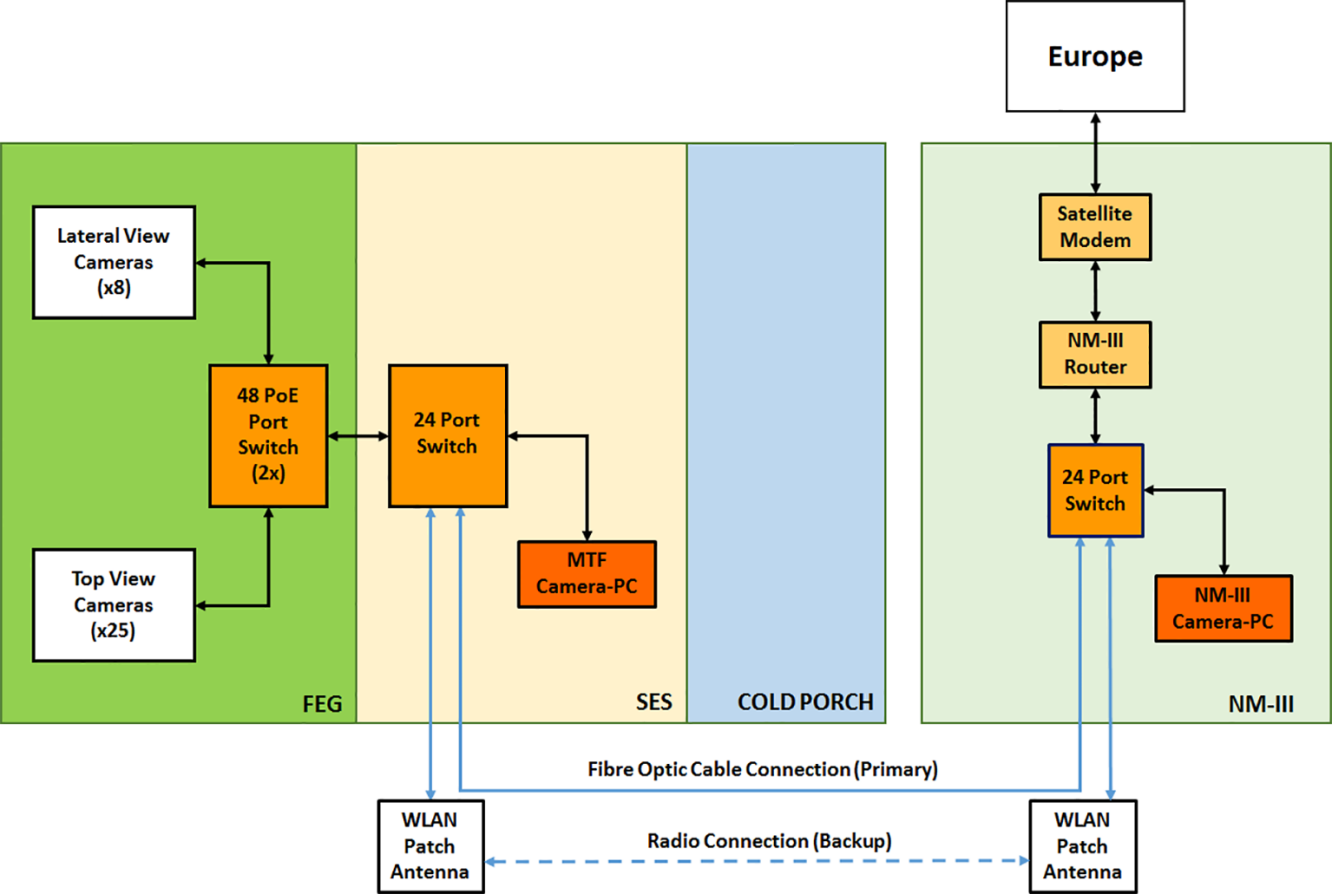
HD color camera network layout, including communication and storage systems.

The HD Color Imaging System is nominally configured to acquire one image per camera per day. The images are saved in jpeg format and have a maximum size of approximately 400 kB. That is done by a custom developed software application based on the programming language Python. The jpeg format was chosen to enable the data transfer of all images of 1 day to Europe using the AWI satellite connection with a very low data rate of 100 kbps. The quality loss of images in bmp format compared to the ones in jpeg format assessed by the human eye was negligible. With this software application, it is possible to schedule the acquisition of daily images (also more than one per day) from the top and lateral view HD color cameras. The images are automatically stored on the PC dedicated for the camera system inside the SES using the following naming convention: HDCam_ < cameraposition > _ < date > _ < time>, with the date expressed in yearmonthday (yyyymmdd), and the time expressed in hourminute (hhmm). A folder/directory structure has been created in order to save the images in separate folders based on camera position. **Figure 7** provides a pictorial view of the camera network.

### Description of the Dual Wavelength Spectral Imaging System

The Dual Wavelength Spectral Imaging System allows the FEG to investigate the use of differential wavelength images for monitoring plant health. The extensive control capabilities of the Illumination System enables the use of approaches such as Normalized Difference Vegetation Index (NDVI) in the evaluation and monitoring of plant health. In particular, the modified GoPro cameras of the Dual Wavelength Spectral Imaging System allow NDVI and NDVI-like approaches to be taken using single RGB images and established post-processing algorithms (Beisel et al., 2018). Using these simple RGB images and commercial cameras allowed the flexible deployment of NDVI imaging within the physical and cost constraints of the project, and minimized the data management loads compared to the use of a true hyperspectral imaging system.

**Figure 8:**
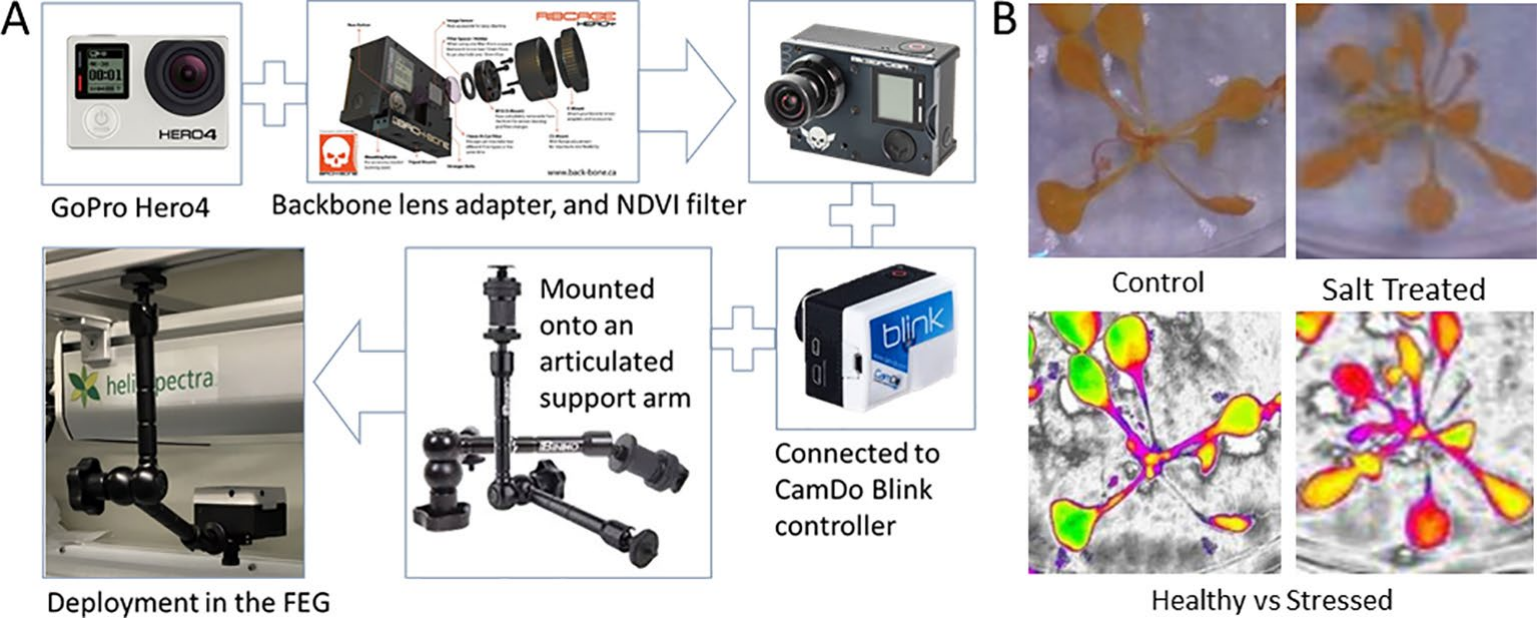
The basic steps involved in the assembly and deployment of the Dual Wavelength Spectral Imaging GoPro system **(A)**, and a test sample of images captured in healthy and salt-stressed plants, including false color NDVI renditions that use color to highlight NDVI pixel values that change with salt stress as an example **(B)**. (Beisel et al., 2018). The imaging filter is NDVI-7 (https://www.irmods.com/ag-ndvi-7) and the camera adaptations were from Ribcage (https://www.back-bone.ca/product/ribcage-air-hero4-mod-kit/). GoPro, Ribcage, and CamDo images taken from the company websites.

For the initial deployment of spectral imaging within the FEG, GoPro Hero4 cameras were modified to become Dual Wavelength Spectral Imagers (Beisel et al., 2018). The infrared (IR) filters were removed to allow Near IR (NIR) into the sensor (**Figures 2** and **8**) and a dual wavelength filter was added so as to collect and segregate the NIR (690–790 nm) and Blue (380–550 nm) wavebands. The filter blocks all greenish-yellow, yellow, and orange wavelengths offering clear separation of the informative Blue and NIR wavelengths to the image sensor. The modifications are enabled through the use of the Back-Bone Ribcage modification kit. The resulting images can be easily color-separated using plugins for ImageJ or other software, and the separated images can be manipulated by pixel math to produce normalized differential images using a wide range of pixel mathematics. Using a single sensor and single filter keeps the visible and NIR images in perfect register, eliminates the need for a mechanical filter wheel in the camera, and keeps the number of images that have to be managed by the data system to a minimum. **Figure 8** shows a summary of the Dual Wavelength Spectral Imaging System setup.

In the 2018 Antarctic season UF monitored plant health with the two modified GoPros, which were mobile and could be placed at any position above the plants. Nevertheless, the position of the GoPros was fixed above one plant tray for a full plant growth cycle to obtain comparable pictures of the plant development. The schedule for image acquisition was controlled by a hardware modification using Blink adapters on the back of the GoPros. Two times a day images were taken automatically by each camera, one during day under nominal lighting conditions and the other one under special lighting conditions, mentioned previously, which enhance the NIR illumination.

After acquisition, each image was directly transferred to the PC dedicated to the camera system inside the SES and stored, in jpeg format, in associated folders in order to have the images saved per camera. The images had a maximum size of approximately 3,000 kB. Also for the images of this type of imaging system, a naming convention was created: UFImager_ < date > _ < time > _*, with the date expressed in yearmonthday (yyyymmdd), and the time expressed in hourminute (hhmm). The images were used in laboratory analyses, as well as being archived in NASA’s LSDA, and used for educational and public outreach purposes. The naming convention facilitates third party analysis and acquisition of the images from the LSDA.

## Remote Support

The Plant Health Monitoring System inside the FEG periodically evaluates the state of the plants and diagnoses possible diseases in early stages. That allows for timely activation of corrective actions to mitigate the impact on the individual plants or even on the entire crop. For that reason, the Plant Health Monitoring System has been implemented with the objective of collecting information suitable for analysis by a knowledge system (either local or remote) to assess and advise prophylactic measures to support the on-site operator. The system provides the following capabilities:

- Immediate warning to the grower about crop failure.
- Support in operations planning.
- Timely feedback about crop performance.

### Description of the University of Wageningen Tasks

The Unit Greenhouse Horticulture of Wageningen Research developed an automated system for remote plant health monitoring, based on the HD Color Camera System described above. The monitoring system:

- Warns the on-site operator of anomalies that may have shown up and advises on mitigating measures (hard warnings).
- Predicts harvest day by fitting a logistic curve through the daily number of pixels classified as leaves by the segmentation and recognition algorithm.
- Evaluates the crop performance to elicit possible revision of climate settings or nutrient supply (soft warnings).

The system makes use of the 24 top view cameras, although in principle it can also be applied to the lateral view images. No camera calibration and no image rectification were applied and for this reason the images presented an evident barrel distortion (any correction for distortion was expected to have a minor effect on prediction of the crop performance). No filters were used and default settings for the white balancing were applied, which were sufficient for the segmentation.

The image of each tray (either one or two in an image; see **Figure 6**) was divided into four sectors (roughly, but not necessarily, one per plant) and the Region of Interest (ROI) in each one, the pixels containing healthy plant material, was selected by an algorithm based on the RGB pixels. A first pre-segmentation of the colored image in the RGB color space based on threshold of the green channel and, in some cases, such as for the lettuce cultivar Outredgeous (*Lactuca sativa* L., type red Romaine, cv. “Outredgeous”), also the red channel, was performed to isolate the crop from the background.

Two types of tray covers were used depending on the cultivation method: gray-colored plastic covers with holes holding various amounts of rockwool blocks depending on the plants cultivated inside the tray, e.g., for tomato or lettuces, and metal covers with small splits for line cultivation on growth mats for the various herbs grown in the FEG such as basil or chives. The tomatoes and peppers were planted in specially designed black 3D printed growth support structures and then located in the trays with holes. The edges of the black support structures were reflective and a reflection of the LED panel was present in some cases for a few days until the crop was big enough to cover the black edges. The metal cover presented relevant reflection of light and, in some cases, of the crop itself. Therefore, the pre-segmented image was transformed into hue–saturation–value (HSV) color space, and the hue and the saturation channel were used to cope with the light reflection on the tray cover and partially on the leaves. Saturation changes were considered by the automatic monitoring system by using dynamic thresholds based on 30% and 90% of the pixels and on 5% and 95% of the pixels corresponding to the area of the saturation channel and the hue channel, respectively.

The distance between tray and LED was constant during cultivation, so it decreased with crop growth and light reflection changed when leaves were very close to the LED. From about 10 cm the color quality of the images rapidly decreased. This was compensated using the texture measures and edge detection of the image in gray format to extrapolate the fine structures of the leaves. To detect leaf discoloration and tomato flowers, the difference of the red and green channel was used. The RGB threshold to be applied for each tray was based on the crop information that the on-site operator manually recorded in a shared file, which listed the status of each plant growth tray (Alfonso et al., 2019), e.g., what plant is located in a given tray and when a crop was moved from the nursery to the production trays.

**Figure 9:**
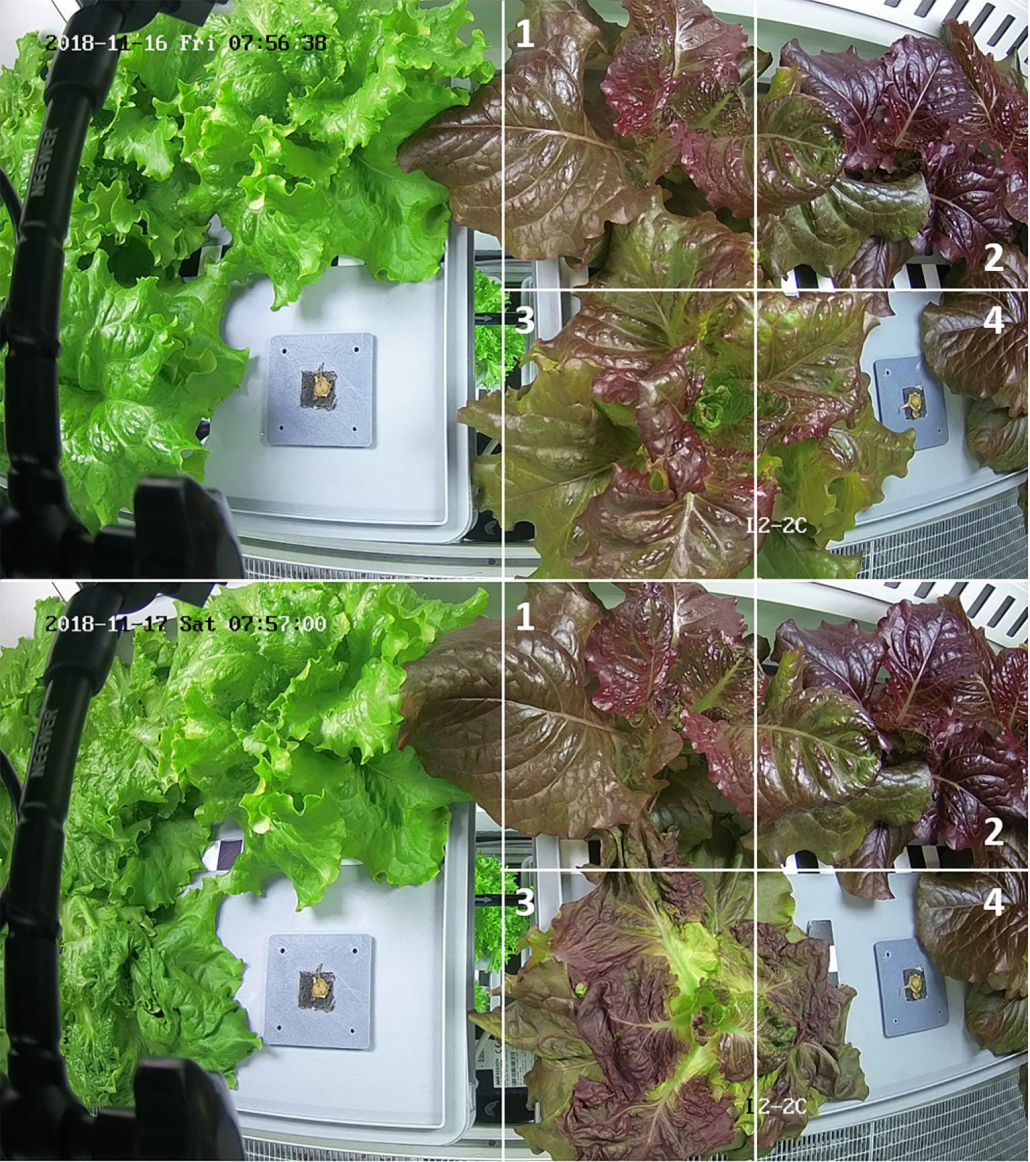
Images of consecutive days of the two trays monitored by the camera L2-2C; for sake of the simplicity only the sectors of the right tray are drawn. The artificial mistreating of the plant leaves in sector three causes a warning. Note that, in this particular case, no plants are growing in sector four of the right and the left tray.

The evolution in time of the ROI in each sector of the daily RGB images was used for the early detection of anomalies. The total number of segmented leaf pixels was compared to the pixels of the previous day. When the size of the ROI decreased by an amount exceeding a preselected threshold of 95% a hard warning was generated by sending an automated e-mail to a human expert, including a description of the detected anomaly and a link to the images leading to the warning (actual and previous) (see **Figure 9**).

The rate of increase of the ROI was used to calculate a growth curve. The parameters of the growth curve were refined daily. Expected harvest time and a crop performance indicator to evaluate crop performance were extracted from it. The latter was done on a daily basis through comparison of the latest predicted maximum growth rate and harvest day to the maximum growth rate and harvest day of the best performing previous cycle of the same crop. If the performance was not satisfactory (either lower growth rate or later harvest), a soft warning was sent out by e-mail and the decline in performance was mentioned (as %) as well as a plot of the extrapolated growth (and the previous, better one) was included.

### Description of the University of Florida Tasks

NDVI is one of the most heavily utilized indices of plant photosynthetic activity and health, and the approach has a long and deep history of data capture and analytic procedures to draw upon. However, NDVI-like approaches are a new area of science when applied to highly controlled environments such as the EDEN ISS MTF, where photons of input and output can be regulated and studied. Thus, application of NDVI-like approaches is an active research area for discoveries related to monitoring plant health in highly controlled environments. Within the FEG and EDEN ISS concept of operations, NDVI and NDVI-related indices provide plant health monitoring that complement morphology, size, and color, and are being investigated as early warning measures of plant stresses.

**Figure 10:**
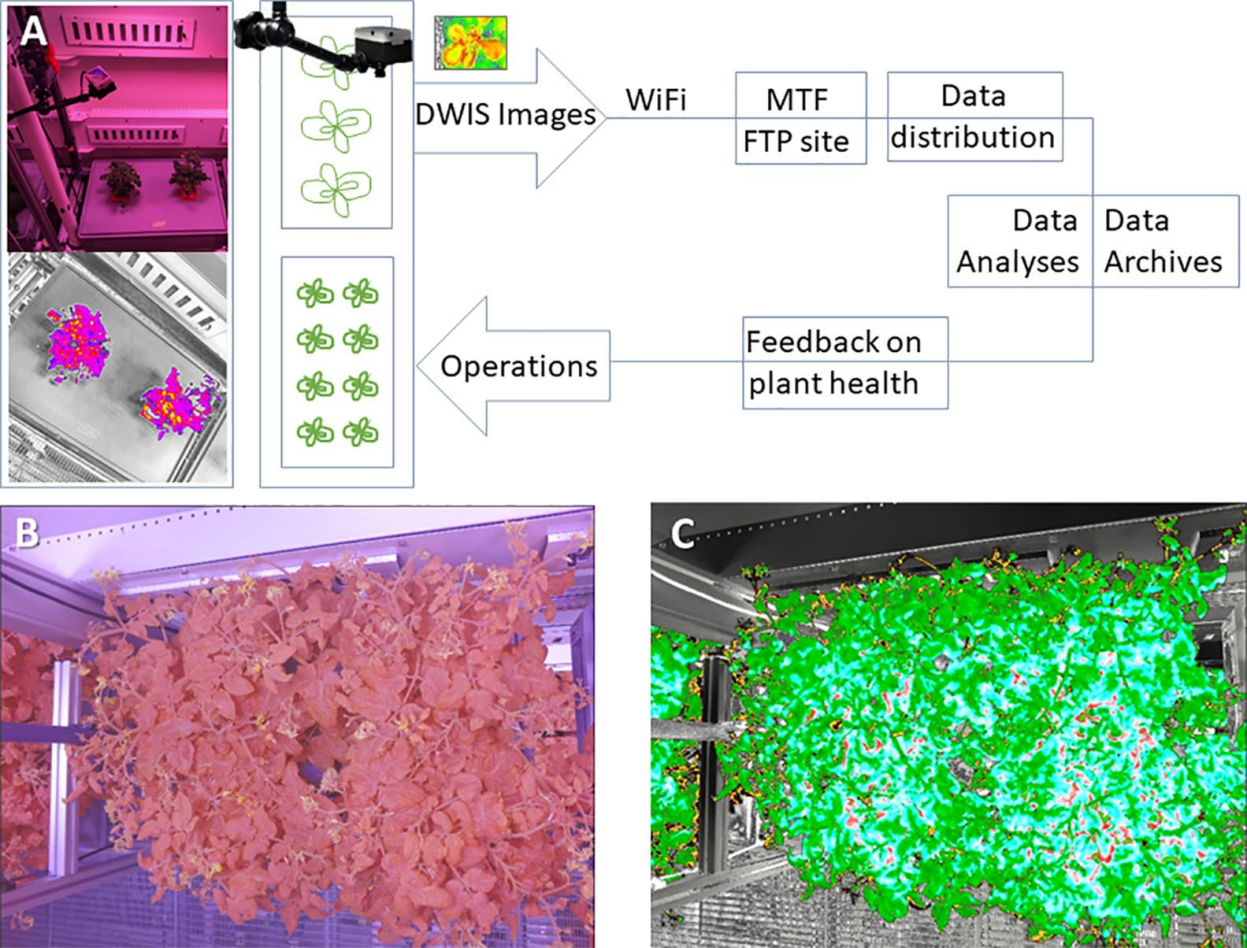
**(A)** The basic information path that was used for the Dual Wavelength Spectral Imaging System (DWIS) integrated into the FEG. **(B)** An example of an unprocessed image taken by the Dual Wavelength Spectral Imaging System over the tomato growth tray. The filter in the imager removes most of the green wavelength from the image. **(C)** A false colored NDVI processed version of the image in **(B)**. The false coloring is provided by a Look Up Table for the NDVI pixel values. The Look Up Table can be varied to adjust the visual presentation of the processed values within each pixel (Beisel et al., 2018). In this example, all of the leaves are essentially healthy and this example shows the range of NDVI pixel false-color values in healthy tomato plants.

The operational model is that Dual Wavelength Spectral Imaging System NDVI data provide an additional layer of plant health monitoring, which would then inform operators of any potential areas of concern and provide feedback to the operations of the FEG (see **Figure 10A**) (Beisel et al., 2018). The raw, unprocessed images are typical RGB images yet, because of the filtering, the images lack all green coloration (see **Figure 10B**). The images are then processed for NDVI and false coloring to assist in analysis (see **Figure 10C**).

## Results After 1 Year of Operations

### HD Color Imaging System

The first year of EDEN ISS plant growth operations was smooth and did not involve serious problems. In fact, most of the warning emails were caused due to issues in the punctual update of the shared plant tray status file by the on-site operator (e.g., following harvest events). This manual procedure could be improved during the next years of operations. A few of the other warnings were caused by natural causes, such a varying orientation of leaves (which can significantly affect the ROI, particularly in young plants), and could easily be dismissed by the experts. Similar effects can be caused due to removal of leaves by cultivation activities. Nevertheless, the hard warning system was accurate on the one failure that was artificially generated (see **Figure 9**). However, the frequency of one image per day is probably too low for on-time response of leafy crops.

The prediction of harvest time did not prove useful, as a trained on-site operator was in the greenhouse (almost) daily and could assess this by himself. Nevertheless, it could be seen that 1 week after transplanting, harvest day could be predicted within ± 2 days. Prediction of harvest time may be more useful whenever an untrained crew has to plan activities in the greenhouse.

**Figure 11:**
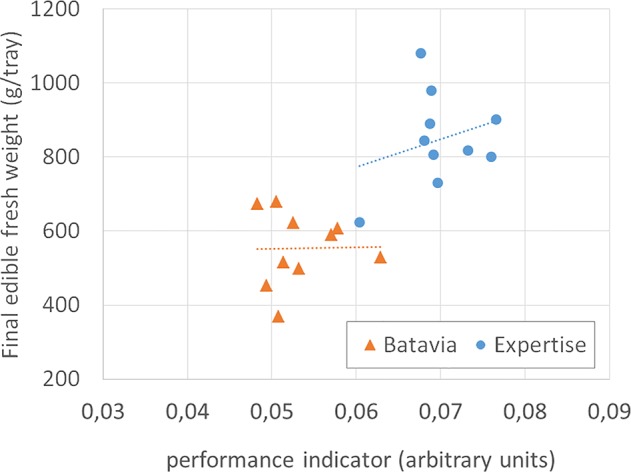
Final edible fresh weight (g/tray) of two cultivars of lettuce, vs the performance indicator, for all cycles long 37 through 39 days. The dotted lines are the linear best fit. The indicator cannot explain the variation in final weight of *Lactuca sativa* L., type Batavia, cv. “Othilie,” and rather poorly of *Lactuca sativa* L., type Crispy Green, cv. “Expertise.”

The correlation between the crop performance indicator and final yield has been analyzed only on lettuce, with mixed results. There was much variation in the final fresh weight for lettuce, whose best predictor proved to be the variation in crop duration (time lapse between transplanting and harvest, caused by the busy schedule of the on-site operator), rather than our indicator. Nevertheless, even for a subset of cycles without correlation between yield and crop growth cycle duration (37 through 39 days), the variation in fresh weight (15%) was larger than the variation in our indicator (6%, **Figure 11**). Clearly, there is a need to evaluate further (and possibly re-define) the performance indicator.

As our limited dataset did not contain enough examples to effectively train a deep-learning algorithm (Birrell et al., 2019), the classical image processing algorithm that we applied did not need images in its training process, as it was based on a fixed set of handcrafted features for its image segmentation. However to cope with situations, such as fruit detection hindered by partial leaf occlusion and flower detection due to flower orientation and occlusions, a Mask R-CNN should be applied. A camera calibration and image rectification will enhance the image quality and allow more accurate measurements of some features such as the flower dimensions. A diffuse illuminance and the use of non-reflecting materials should be used to reduce the reflections.

### Dual Wavelength Spectral Imaging System

The Dual Wavelength Spectral Imaging System proved to be a largely reliable imaging system within the FEG, returning to the project typically two images per day from each of the two imagers. Because each of the imagers was independently controlled by software settings within the camera itself, there were some operational tendencies that suggest improvements for the next deployment. For example, the clock settings within the GoPros tended to drift from the clocks within the FEG, prompting needs for operator resets of the imagers. However, the concept of having a movable imager that can be positioned at various places within a large growing system proved to be a success in that several growth systems were imaged over the course of the year.

**Figure 12:**
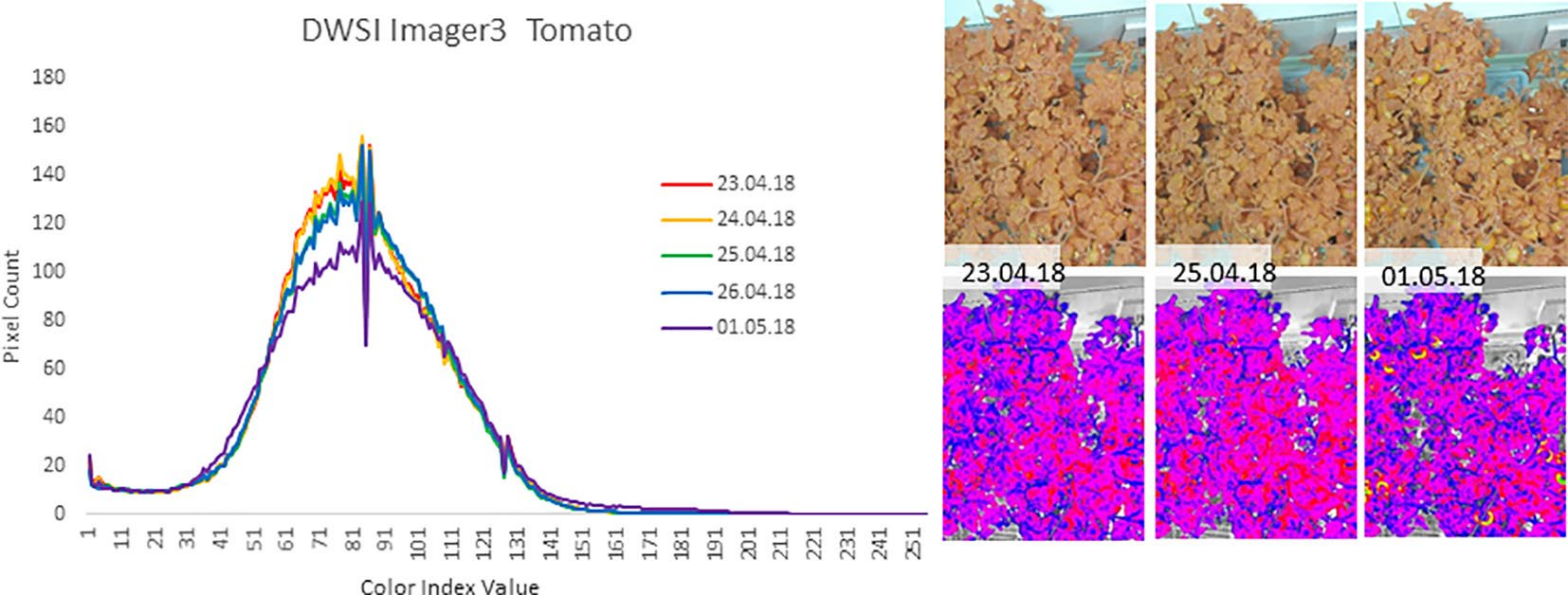
An example of plant suboptimal health indications from May of 2018 where the Dual Wavelength Spectral Imaging System (DWIS) recorded an anomaly in the health of one of the trays of tomato plants in the FEG. The graph depicts the count of pixels within the image of a particular NDVI value (Beisel et al., 2018). The Y axis is the number of pixels of each NDVI value, normalized to a range from 1 to 256. From April 23 to 25 2018, the pixel values begin to drop and shift to higher NDVI values and reach a maximum change near May 1. The images to the right show portions of the unprocessed image (top) and the corresponding false color NDVI image (bottom) for each of the given imaging days. The graphs were obtained from equal regions of interest out of each NDVI image such that non-plant parts of the image were not included in the pixel value counts.

The FEG proved to be an effective operational test-bed for using NDVI for monitoring plant health. For example, in late April and early May of 2018, there was an unrecognized problem in the Nutrient Delivery System for one of the trays of tomato plants. From the color cameras, there was no discernable difference in the appearance of the tomato plants, but the Dual Wavelength Spectral Imaging System NDVI images showed a quantitative decline in plant health preceding overt visual indications by several days (see **Figure 12**). The changing of the NDVI values preceded the visual notification by the crew by 3–5 days. When the nutrient solution was replaced, the NDVI images returned to normal. These observations indicate that there is value to NDVI imaging in operational production spaces, and suggest further study on the applicability of NDVI to other stresses such as plant disease and infestation.

## Data Availability Statement

The datasets generated for this study are available on request to the corresponding author.

## Author Contributions

PZ was the on-site operator of the EDEN ISS greenhouse in Antarctica during the experimental campaign, which means he was responsible for plant cultivation and overall system maintenance. CZ, VV, MB, MD, and DS assisted in the conduct of the experimental campaign from the Mission Control Center in Germany providing technical support and horticulture expertise. CZ was the main responsible person for the design and setup of the EDEN ISS ground network. He installed the hardware as well as the data distribution software for the HD Color Imaging System and Dual Wavelength Spectral Imaging System into the Mobile Test Facility. He was responsible for maintaining the system and was the troubleshooting point of contact. He contributed the descriptions of HD Color Imaging System and Dual Wavelength Spectral Imaging System to the paper. MB provided input on the abstract and introduction. VV worked on the EDEN ISS Mobile Test Facility Infrastructure section. VV, MB, and CZ did extensive reviewing of the overall paper. DS was the project manager of EDEN ISS. CS, FK, EM, AM, and G-JS were responsible for the definition of the cultivation plan and supported all crop cultivation-related issues. They processed and analyzed the images of the HD Color Imaging System to detect anomalies and evaluate crop performance. They contributed to the paper by describing the analysis process of the HD Color Imaging System and the corresponding results. AC, RF, and DDS (Telespazio) were responsible for the HD Color Imaging System. They developed a custom data distribution software application and hosted the camera test platform to provide the needed support for system configuration and troubleshooting. AC described the user home bases in the paper and worked on the description of the HD Color Imaging System. A-LP and RJF (University of Florida) were on site at the EDEN ISS for installation and analysis of Dual Wavelength Spectral Imaging System, performed the NDVI data analysis, and contributed these analyses to the manuscript.

## Funding

The EDEN ISS project has received funding from the European Union’s Horizon 2020 research and innovation program under grant agreement No 636501. The participation of the Dutch team was co-financed by the Top Sector Horticulture & Starting Materials. The participation of the UF team was funded by NASA grant 80NSSC17K0199 and the Florida Space Grant Consortium grant FSGC NNX15_016 FSGC08 170829.

## Conflict of Interest

Authors AC, RF, and DDS were employed by the company Telespazio S.p.A. 

The remaining authors declare that the research was conducted in the absence of any commercial or financial relationships that could be construed as a potential conflict of interest.
